# Screening for Liver Steatosis and Fibrosis in Patients with Inflammatory Bowel Disease Using Vibration Controlled Transient Elastography with Controlled Attenuation Parameter

**DOI:** 10.3390/jcm11195959

**Published:** 2022-10-09

**Authors:** Anca Trifan, Remus Stafie, Adrian Rotaru, Ermina Stratina, Sebastian Zenovia, Robert Nastasa, Laura Huiban, Tudor Cuciureanu, Cristina Muzica, Stefan Chiriac, Irina Girleanu, Ana-Maria Singeap, Catalin Sfarti, Camelia Cojocariu, Oana Petrea, Carol Stanciu

**Affiliations:** 1 Department of Gastroenterology, Grigore T. Popa University of Medicine and Pharmacy, 70015 Iasi, Romania; 2 Institute of Gastroenterology and Hepatology, “St. Spiridon” University Hospital, 700111 Iasi, Romania

**Keywords:** inflammatory bowel disease, non-alcoholic fatty liver disease, liver fibrosis, vibration-controlled transient elastography

## Abstract

Background and Aims: Inflammatory bowel diseases (IBD) are frequently associated with extraintestinal manifestations, hepatic injury being of concern in these patients. Current literature reports an increased prevalence of liver steatosis and fibrosis in subjects with IBD and the pathophysiology is yet to be completely understood. The aim of this study was to assess the prevalence of non-alcoholic fatty liver disease (NAFLD) in patients with IBD, as well as to determine the factors that connect these two disorders. Methods: From September 2021 to June 2022, 82 consecutive IBD patients were enrolled from a tertiary care center hospital in Iasi. Vibration-Controlled Transient Elastography with Controlled Attenuation Parameter (CAP) was used to assess the presence of NAFLD, with a cut-off score for CAP of 248 dB/m. Significant liver fibrosis was considered at a cut-off for liver stiffness measurements (LSM) of 7.2 kPa. Results: In total, 82 IBD patients (54.8% men, mean age of 49 ± 13 years) were included, 38 (46.3%) of them being diagnosed with NAFLD, with a mean CAP score of 286 ± 35.4 vs. 203 ± 29.7 in patients with IBD only. Age (*β* = 0.357, *p* = 0.021), body mass index (BMI) (*β* = 0.185, *p* = 0.048), disease duration (*β* = 0.297, *p* = 0.041), C—reactive protein (*β* = 0.321, *p* = 0.013), fasting plasma glucose (*β* = 0.269, *p* = 0.038), and triglycerides (*β* = 0.273, *p* = 0.023) were strongly associated with the presence of liver steatosis. The multivariate analysis showed that older age, BMI, and disease duration were strongly associated with significant liver fibrosis in our group. Conclusions: NAFLD is a multifaced pathology with growing prevalence among IBD patients. Additional studies are needed to completely understand this problem and to create a solid evidence-based framework for more effective preventative and intervention strategies.

## 1. Introduction

Inflammatory bowel diseases (IBD), with ulcerative colitis (UC) and Crohn’s disease (CD) being the main forms, are caused by a dysregulated immune response in the hosts, favored by genetic susceptibility. In addition to symptoms related to the digestive tract, about 40% of patients with IBD also have extraintestinal manifestations, of note being those related to the hepatobiliary tract. Studies report a various number of hepatobiliary manifestations in patients with IBD, 5% of them developing chronic liver disease [1,2,3].

Chronic liver disorders (CLDs) have a significant influence on global health care systems. Non-alcoholic fatty liver disease (NAFLD) and alcohol-related liver disease (ALD) continue to be the two most prevalent liver diseases worldwide among CLDs [4]. Moreover, they share a similar spectrum of fatty liver/steatosis, steatohepatitis, fibrosis, cirrhosis, and hepatocellular carcinoma (HCC) despite diverse risk factors being involved [5]. As previously described, due to several susceptibility characteristics, long-term heavy alcohol users continue to be at risk for severe liver disease, including alcoholic steatohepatitis (ASH), cirrhosis, and HCC [6]. NAFLD, on the other hand, is characterized by the presence of excessive fat in more than 5% of the hepatocytes in patients with low alcohol consumption and no other causes of liver disease. Patients may advance from a benign stage of excessive hepatic steatosis or non-alcoholic fatty liver (NAFL) to the more aggressive subtype, namely steatohepatitis (NASH), which has an important progressive nature, leading to end-stage liver disease and HCC [7,8,9]. Due to the growing number of patients with IBD and the availability of new diagnostic technologies, it has been hypothesized in recent years that this illness may potentially be related to NAFLD, with an incidence three-fold higher than that of the general population [10,11].

Although the exact causes of NAFLD and the factors contributing to the progression to end-stage liver disease are still not fully understood, it has been proven that certain conditions can increase the risk of developing this pathology [12]. Classic risk factors such as obesity, hypertension, insulin resistance and dyslipidemia can also be present in patients with IBD [13]. Some studies indicate that patients with IBD are prone to developing NAFLD, due to chronic inflammation, digestive alterations, previous surgeries and changes of the fecal microbiota [14,15]. In addition, certain drugs regularly used for treating IBD can also cause liver damage, such as steroids, anti-tumor necrosis factors, and azathioprine [16,17].

Previous studies report an increased prevalence of NAFLD in IBD patients, ranging from 1.5% to 55%, when compared to the general population [18,19,20]. This discrepancy is mainly related to differences in the diagnostic method, definition of the disease, and the study population [21].

In the diagnosis of NAFLD, liver biopsy still represents the gold standard method but, given the invasive nature, increased costs, high rate of possible errors, and also the possibility of life-threatening complications during the procedure (bleeding, pneumothorax, hemothorax, hemobilia, biliary peritonitis, intestinal perforation, or even death), it is rarely used in clinical practice [22,23]. As a result, non-invasive methods are preferred, such as ultrasonography and Vibration-Controlled Transient Elastography (VCTE) with Controlled Attenuation Parameter (CAP) [24,25]. VCTE with CAP is an easy and fast method to perform and allows the simultaneous assessment of both liver fibrosis and steatosis, with low failure rates and good sensitivity and specificity [26,27].

Bearing in mind that the mortality rate is higher in patients with IBD and NAFLD than in those with IBD alone, the evaluation of liver steatosis and fibrosis is crucial in this group of subjects [28]. Therefore, it is essential to understand the causes and mechanisms that can result in liver fibrosis and hepatic steatosis in IBD patients, in order to avoid this condition from occurring.

The aim of our study was to assess the prevalence and severity of NAFLD in patients with IBD using VCTE with CAP. In our group of patients, we additionally examined biochemical alterations and other risk variables related to NAFLD.

## 2. Materials and Methods

### 2.1. Patients

We prospectively enrolled 82 consecutive patients with an established diagnosis of IBD from a tertiary referral hospital in north east of Romania, who were evaluated between September 2021 and June 2022. Clinical and biochemical data and personal medical history, including comorbidities, were collected. VCTE with CAP was performed for each patient, who were divided into two groups, namely IBD with NAFLD and IBD only, accordingly.

The inclusion criteria were as follows: (1) age over 18 years, (2) established diagnosis of UC or BC, (3) signed the informed consent form, and (4) reliable transient elastography examination. Subjects with (1) other known cause of liver disease (chronic viral hepatitis, primary biliary cholangitis, autoimmune hepatitis, liver neoplasia) or (2) history of alcohol abuse (more than 10 g/day for women and 20 g/day for men) were excluded. The time since the diagnosis, current treatment, disease extension and severity were also recorded for all individuals. This study was approved by the Ethics Committee of our University and was conducted according to the principles of the Declaration of Helsinki. Each participant signed a written informed consent form.

### 2.2. Vibration Controlled Transient Elastography with Controlled Attenuation Parameter

VCTE with CAP was performed using a FibroScan^®^ 502 Touch (EchoSens, Paris, France) by two experienced physicians who had conducted over 300 examinations. In summary, the examination was carried on patients after a minimum of four hours of fasting, first using the standard M probe, changing to the XL probe according to the device’s indications, with the tip of the transducer being placed in the 9th to 11th intercostal space, corresponding to the right hepatic lobe [29,30]. A measurement was considered to be valid if ten readings with a mean interquartile range lower than 30% were obtained [31]. Liver stiffness measurements (LSM) were collected in kilopascals (kPa) using specific cut-off values corresponding to different stages of fibrosis, namely ≥5.6 kPa for mild fibrosis (F1), ≥7.2 kPa for significant fibrosis (F2), ≥9.7 kPa for advanced fibrosis (F3), and 12.5 kPa for liver cirrhosis (F4) [24,32,33]. CAP values were expressed in decibels/meter (dB/m), with the following cut-off values: ≥248 dB/m for mild steatosis (S1), ≥268 for moderate steatosis (S2), and ≥280 for severe steatosis (S3) [34].

### 2.3. Statistical Analysis

IBM SPSS, Version 22.0 was used to calculate descriptive statistics for all factors (IBM SPSS Inc. Chicago, IL, USA). The Kolmogorov–Smirnov test was used to determine if the distribution of numerical variables was normally distributed. Continuous variables were expressed as mean and standard deviation or percentage. Statistical analysis included Student’s *t*-test for comparison of two means for parametric data and the Mann–Whitney U test for comparison of two means for non-parametric data. Univariate and multivariate linear regression were performed to identify variables significantly associated with the presence of hepatic steatosis or significant liver fibrosis. All variables with *p*  <  0.05 in the univariate analysis were included in multivariate analysis.

## 3. Results

### 3.1. Patients Characteristics

A total of 82 IBD patients met the inclusion criteria and were included in the final statistical analysis. Patients were divided into two groups, namely patients with IBD (53.7%) and those with both IBD and NAFLD (46.3%). Disease characteristics of IBD patients are presented in Table 1. UC was present in 54.9% of patients, whereas 45.1% of them suffered from CD. Most patients with UC had left-sided colitis, accounting for almost half of them (42.3%). Regarding CD, ileal localization was the most frequent one (45.9%), inflammatory behavior being the most prevalent among this group of patients (62.2%). Almost all patients who underwent the VCTE with CAP examination were in the remission phase of the disease. A small majority of subjects were males (54.8%), with a mean age of 49 years for the whole cohort.

### 3.2. Clinical and Biochemical Profile of Subjects with IBD versus IBD with NAFLD

The patients’ characteristics are summarized in Table 2, with both clinical and biochemical data being recorded in order to further emphasize the differences between IBD patients and subjects with IBD and NAFLD. The majority of patients in our study were normal weight and overweight, but with a significant difference between the two groups of subjects regarding mean body mass index (BMI) value (26.3 ± 3.8 vs. 22.9 ± 4.1, *p* < 0.001).

Patients with NAFLD presented a more important inflammatory syndrome, with higher CRP (*p* = 0.041) and ferritin (*p* = 0.012) levels, but with no significant statistical difference between leukocytes levels (*p* = 0.194). Moreover, similar to the general population, individuals with IBD and NAFLD had higher values of fasting plasma glucose (*p* < 0.001), when compared to their non-NAFLD counterparts, as well as a higher prevalence of type 2 diabetes mellitus (T2DM) (*p* < 0.001). Regarding lipid metabolism, there was no significant statistical difference between the two groups of patients when total cholesterol levels were evaluated. On the other hand, patients with NAFLD presented higher values of triglycerides (*p* = 0.037) and lower values of high-density lipoprotein cholesterol (HDL-c) (*p* < 0.001). In liver enzymes, a significant statistical difference was observed between the aspartate aminotransferase (AST)levels (*p* = 0.003). Regarding disease related data, patients with NAFLD were older at diagnosis (*p* = 0.027) and also had a longer disease duration (*p* = 0.031).

### 3.3. Comparison of CAP and LSM Values between the Two Groups

In our study, the diagnosis of hepatic steatosis, defined by a CAP ≥ 248 dB/m, was confirmed in 38 subjects. The mean CAP score in the overall cohort was 251 ± 51.8 dB/m, with a significant statistical difference between the two study groups, i.e., 286 ± 160 35.4 dB/m for those with NAFLD, and 203 ± 29.7 dB/m for those without NAFLD (*p* < 0.001).

The same pattern was observed when mean values of LSM were analyzed, with 6.8 ± 1.9 kPa for the NAFLD and IBD group and 5.1 ± 1.1 kPa for individuals with only IBD (*p* = 0.017). Furthermore, the majority of patients had no or mild liver fibrosis, while 21 (25.6%) of them were diagnosed with at least significant hepatic fibrosis.

### 3.4. Factors Associated with Liver Steatosis and Significant Fibrosis

We conducted in our study a univariate analysis, followed by multivariate linear regression analysis, in order to identify the variables significantly associated with the presence of liver steatosis and significant fibrosis in patients with IBD and NAFLD (Table 3). The multivariate analysis showed that age (β = 0.357, *p* = 0.021), BMI (β = 0.185, *p* = 0.048), disease duration (β = 0.297, *p* = 0.041), CRP (β = 0.321, *p* = 0.013), fasting plasma glucose (β = 0.269, *p* = 0.038), the presence of T2DM (β = 0.215, *p* = 0.033), and triglycerides (β = 0.273, *p* = 0.023) were strongly associated with hepatic steatosis. The age at diagnosis and history of bowel resection were strongly correlated with liver steatosis in the univariate analysis, but with no correlation in the multivariate analysis.

Regarding liver fibrosis, the multivariate analysis revealed that older age (β = 0.224, *p* = 0.012), BMI (β = 0.242, *p* = 0.019), and disease duration (β = 0.293, *p* = 0.038) were strongly associated with significant liver fibrosis in our cohort. Moreover, we also found a significant negative association between platelet count and higher values of LSM (β = −0.134, *p* = 0.042). Even though, in the multivariate analysis, there was no significant correlation, male gender, AST and triglycerides levels were still associated with significant liver fibrosis in the univariate analysis. Of note is the fact that patients with higher BMI had an increased risk for significant liver fibrosis (OR 1.9, 95% CI 1.24–2.87, *p* = 0.027).

## 4. Discussion

As far as we know, this is the first prospective study in Romania to focus on screening for liver steatosis and fibrosis in a cohort of IBD subjects, in order to evaluate the prevalence of NAFLD in this special group of patients and to further understand the associated risk factors.

NAFLD has recently become the most widespread form of chronic liver disease in the world, affecting 25% of the general population and playing a significant role in the emergence of hepatic cirrhosis and HCC [35,36]. In line with the general population trends, the diagnosis of NAFLD in IBD patients has become more frequent, with a prevalence of about 32% [37]. According to a recent meta-analysis, NAFLD is more common among individuals with severe IBD symptoms, such as prolonged disease duration or a history of abdominal surgery [38].

Fatty liver disease is becoming more common in IBD patients, perhaps as a result of the utilization of cutting-edge diagnostic techniques such as transient elastography as well as due to the changing of IBD phenotypes and the significant increase in IBD-related obesity. There are two broad groups of NAFLD risk factors for IBD patients: those linked to the metabolic syndrome and those linked to intestinal inflammation. These, in turn, indicate two different forms of IBD-related NAFLD, the “traditional” metabolic NAFLD and a more IBD-specific variety with stronger involvement of the microbiota–gut–liver axis and fewer metabolic abnormalities [39].

Previous studies have reported discordant data regarding the prevalence of NAFLD in IBD cohorts, the wide range in prevalence rates most likely being explained by varying study populations and NAFLD diagnostic standards. In line with prior studies, 46.3% of the analyzed patients in our cohort were diagnosed with NAFLD by VCTE with CAP [18,40,41]. Differently, Likhitsup et al. reported a prevalence of 54% using ultrasonography as the diagnosis method [42]. Moreover, earlier studies using liver biopsy revealed a prevalence of hepatic steatosis of up to 88% in patients with IBD [18,43]. On the other hand, previous studies by Glassner et al., Principi et al., and Fousekis et al. reported lower prevalences of NAFLD, namely 13.3%, 28%, and 20%, respectively [20,44,45]. It is important to note that the low prevalence found by Fousekis et al. may be due to the region’s unique dietary customs, as the Mediterranean diet has been related to a decrease in liver steatosis [46].

In our cohort, older age was found to be independently correlated with NAFLD, which could be attributed to the steady increase of metabolic risk variables with aging. The association between the existence and severity of steatosis and the natural history of IBD has been the subject of conflicting information. Similar to our results, Glassner et al. reported that disease duration, disease activity, and prior surgery are associated with the development of NAFLD, and these data have been confirmed by other researchers [10,20,47,48]. In line with our findings, Yen et al. and Veltkamp et al. failed to show a relationship between IBD-related medication and the risk of developing NAFLD [1,49].

According to a recently released meta-analysis, type 2 diabetes, high blood pressure, obesity, insulin resistance, metabolic syndrome, chronic kidney disease, methotrexate use, IBD surgery, and longer disease duration are all risk factors for the development of NAFLD [38].

One of main predictors of NAFLD in our study group was the BMI, being strongly associated with the presence of hepatic steatosis (β = 0.185, *p* = 0.048) and significant liver fibrosis (β = 0.242, *p* = 0.019), even though almost half of the patients with IBD and NAFLD were of normal weight. Regarding the metabolic profile, subjects with NAFLD had higher mean triglycerides and fasting plasma glucose levels and higher prevalence of T2DM, all of these being independently associated with liver steatosis. A recent retrospective study, comparing NAFLD patients with and without IBD, found that those with IBD tended to develop NAFLD with fewer metabolic risk factors. These results indicate that, in addition to the metabolic syndrome, other potential risk factors such as genetic variations may be at work, predisposing patients with IBD to develop NAFLD [48].

As shown in patients with chronic hepatitis C, transient elastography is a well-established, highly accurate, non-invasive approach for measuring hepatic fibrosis [50]. Existing knowledge regarding the occurrence and pathophysiology of hepatic fibrosis in IBD patients is quite limited, despite the fact that liver damage is a common finding in this group of subjects.

In our study, 21 (25.6%) patients had at least significant liver fibrosis, eight (9.7%) of them having advanced hepatic fibrosis, and these findings are consistent with data from a recent meta-analysis [37]. Another previous study also reported similar results, with a higher prevalence of liver fibrosis among patients with both IBD and NAFLD [51]. Although the exact cause of the co-existence of NAFLD and IBD, leading to liver fibrosis, is unknown, it is thought to be influenced by a variety of factors, including metabolic syndrome (MS), microbial dysbiosis, immune activation, drugs, the severity and duration of the disease, and previous surgical interventions [10]. Disease duration was indeed independently associated, in our study, with the presence of at least significant liver fibrosis (β = 0.293, *p* = 0.038), results that are similar to those reported in previous publications [1,52]. Patients with IBD are subject to several NAFLD risk factors, such as hepatotoxic medications, chronic relapsing inflammation, and altered gut flora [10]. In our cohort, BMI (β = 0.242, *p* = 0.019) and older age (β = 0.244, *p* = 0.012) were also strongly associated with the presence of hepatic fibrosis in the multivariate analysis, and these results are supported by other authors [51,53].

Care for IBD patients has become increasingly difficult and specialized; as a result, IBD subjects are at risk of not adhering to speciality examination and follow-up for their liver illness, particularly if the disease is subtle and asymptomatic.

The strengths of our study are represented by the prospective design and a well-defined cohort. Furthermore, we conducted a regular screening program for liver disease in a group of patients using a validated and reliable diagnostic method, transient elastography with CAP. The low likelihood of selection bias and assurance of the high incidence of NAFLD we discovered are provided by this study’s methodology. Moreover, as far as we know, this is the first prospective study conducted in Romania in which patients with IBD were screened for NAFLD.

Our study has several limitations that we need to acknowledge. First, the current gold standard method for the diagnosis of liver fibrosis and steatosis still remains liver biopsy, which we did not perform due to the numerous disadvantages. The second potential limitation consists of the small cohort we evaluated, which could lead to limited ability to further investigate other relationships, such as the influence of the treatment on liver steatosis. Thirdly, we did not investigate the genetic variations linked to hepatic steatosis, such as patatin-like phospholipase domain-containing protein 3 (PNPLA3), which may shed more light on the pathophysiology of IBD-related NAFLD. In addition, more relevant results could have been available if we had included in our study a control group, in order to investigate the steatogenic potential of IBD.

## 5. Conclusions

Our findings indicate that the prevalence of NAFLD in IBD patients is indeed high, which is why screening for NAFLD and its associated risk factors may be important in this group of patients, in order to prevent or delay the development of liver disease. Additionally, early diagnosis of NAFLD via VCTE with CAP may have a significant effect on the progression of liver disease.

To fully understand this issue and provide a strong evidence-based framework for more efficient preventative and intervention techniques, additional clinical and pathophysiology investigations are required.

## Figures and Tables

**Table 1:** Disease Characteristics.

Disease Location	
CD, *n* (%)	37 (45.1)
Ileal	17 (45.9)
Colon	5 (13.5)
Ileocolonic	10 (27.1)
Upper gastrointestinal tract	2 (5.4)
Perianal	3 (8.1)
UC, *n* (%)	45 (54.9)
Proctitis	11 (24.4)
Left-sided colitis	19 (42.3)
Extensive colitis	15 (33.3)
CD behavior, *n* (%)	
Inflammatory	23 (62.2)
Stricturing	9 (24.3)
Perforating	5 (13.5)
Ongoing medication, *n* (%)	
5-ASA	43 (52.5)
Corticosteroids	6 (7.3)
Azathioprine	9 (10.9)
Biological agent	24 (29.3)
Proportion in remission at TE reading, *n* (%)	
CD	31 (83.7)
UC	39 (86.6)

CD: Crohn’s disease, UC: ulcerative colitis, 5-ASA: 5-aminosalicylic acid, TE: Transient Elastography.

**Table 2:** Patients Characteristics.

	Overall Cohort	IBD + NAFLD	IBD	*p* Value
Patients’ characteristics	*n* = 82	*n*, (%) = 38 (46.3)	*n*, (%) = 44 (53.7)	
Age (years)	49 ± 13	53 ± 17	47 ± 14	0.001 ^a^
Male sex, *n* (%)	45 (54.8)	24 (63.1)	21 (47.7)	0.158 ^a^
Disease duration, years	6.5 ± 5.1	7.2 ± 4.1	5.4 ± 3.8	0.031 ^a^
Age at diagnosis, years	40.6 ± 12.4	41.3 ± 8.8	38.7 ± 10.1	0.027 ^a^
Bowel resection, *n* (%)	13 (15.8)	8 (21)	5 (11.3)	0.022 ^a^
BMI (kg/m^2^)	25.3 ± 4.7	26.3 ± 3.8	22.9 ± 4.1	<0.001 ^a^
Underweight (BMI < 18.5), *n* (%)	4 (4.8)	1 (2.6)	3 (6.8)	0.241 ^a^
Normal weight, *n* (%)	44 (53.7)	17 (44.7)	27 (61.4)	0.326 ^a^
Overweight (BMI ≥ 25), *n* (%)	25 (30.6)	14 (36.9)	11 (25)	0.512 ^a^
Obese (BMI ≥ 30), *n* (%)	9 (10.9)	6 (15.8)	3 (6.8)	0.339 ^a^
CAP (dB/m)	251 ± 51.8	286 ± 35.4	203 ± 29.7	<0.001 ^a^
Leukocyte number (10^9^/L)	7.4 ± 2.3	7.6 ± 2.1	7.1 ± 1.8	0.194 ^a^
Platelet count (10^9^/L)	247 ±71	248 ± 76	238 ± 65	0.271 ^a^
CRP (mg/dL)	0.51 ± 0.8	0.64 ± 0.69	0.31 ± 1.2	0.041 ^a^
Ferritin level (ng/mL)	138 (89–197)	131.7 ± 7.8	97.2 ± 8.9	0.012 ^b^
Fasting plasma glucose (mg/dL)	86 ± 43	97 ± 22	81 ± 38	<0.001 ^a^
Creatinine (mg/dL)	0.8 ± 0.3	0.9 ± 0.2	0.7 ± 0.1	0.027 ^a^
ALT (IU/L)	43 ± 31	46 ± 20	38 ± 27	0.057 ^a^
AST (IU/L)	35 ± 23	39 ± 14	27 ± 20	0.003 ^a^
GGT (IU/L)	39 ± 37	41 ± 24	37 ± 28	0.122 ^a^
ALP (IU/L)	78 (58–97)	85 ± 26	79 ± 32	0.341 ^b^
Total bilirubin (mg/dL)	0.7 ± 0.3	0.8 ± 0.3	0.7 ± 0.2	0.625 ^a^
Total cholesterol (mg/dL)	146 ± 54	154 ± 39	142 ± 43	0.097 ^a^
Triglycerides (mg/dL)	139 ± 74	147 ± 52	109 ± 61	0.037 ^a^
Albumin (g/dL)	4.6 ± 1.8	4.5 ± 1.4	4.7 ± 1.2	0.297 ^a^
LDL-c (mg/dL)	119 ± 41	121 ± 39	117 ± 34	0.213 ^a^
HDL-c (mg/dL)	46 ±11	41 ± 10	48 ± 11	<0.001 ^a^
Hypertension, *n* (%)	16 (19.5)	12 (31.5)	4 (9.1)	0.021 ^a^
T2DM, *n* (%)	7 (8.5)	6 (15.7%)	1 (2.2%)	<0.001 ^a^
LSM (kPa)	6.08 ± 1.8	6.8 ± 1.9	5.1 ± 1.1	0.017 ^a^
Fibrosis stage, *n* (%)				
F0	36 (43.9%)	14 (36.8%)	22 (50%)	
F1	25 (30.4%)	10 (26.3%)	15 (34.1%)	
F2	13 (16.1%)	8 (21.1%)	5 (11.3%)	
F3	7 (8.4%)	5 (13.2%)	2 (4.6%)	
F4	1 (1.2%)	1 (2.6%)	0 (0%)	

IBD: Inflammatory bowel disease, NAFLD: Non-alcoholic fatty liver disease, BMI: body mass index, CAP: Controlled Attenuation Parameter, CRP: C-reactive protein, ALT: alanine aminotransferase, AST: aspartate aminotransferase, GGT: gamma-glutamyl transferase, ALP: alkaline phosphatase, LDL-c: low-density lipoprotein cholesterol, HDL-c: high-density lipoprotein cholesterol, T2DM: type 2 diabetes, LSM: liver stiffness measurements. F0: no fibrosis, F1: mild fibrosis, F2: significant fibrosis, F3: advanced fibrosis, F4: liver cirrhosis. a—Student’s t-test. b—Mann–Whitney U test.

**Table 3:** Factors associated with hepatic steatosis and significant liver fibrosis using univariate and multivariate linear regression analyses.

	Steatosis				Significant Fibrosis			
Variable	Univariate		Multivariate		Univariate		Multivariate	
	β	*p*	β	*p*	β	*p*	β	*p*
Age	0.216	0.017	0.357	0.021	0.145	0.034	0.244	0.012
Male gender	0.385	0.014	0.122	0.378	0.194	0.009	0.014	0.878
BMI	0.311	0.036	0.185	0.048	0.199	<0.001	0.242	0.019
Disease duration	0.267	<0.001	0.297	0.041	0.211	0.173	0.293	0.038
Age at diagnosis	0.331	0.024	0.188	0.355	0.107	0.284	-	-
Bowel resection	0.291	0.004	0.207	0.412	0.069	0.449	-	-
Leukocyte number	0.118	0.591	-	-	0.192	0.427	-	-
Platelet count (G/L)	0.132	0.201	-	-	−0.255	<0.001	−0.134	0.042
CRP (mg/dL)	0.256	0.029	0.321	0.013	0.117	0.421	-	-
Ferritin level (ng/mL)	0.032	0.841	-	-	0.078	0.391	-	-
Fasting plasma glucose (mg/dL)	0.269	<0.001	0.269	0.038	0.067	0.284	-	-
Creatinine (mg/dL)	0.127	0.273	-	-	0.122	0.291	-	-
ALT (IU/L)	0.129	0.431	-	-	0.161	0.184	-	-
AST (IU/L)	0.351	0.018	0.101	0.413	0.281	<0.001	0.078	0.518
GGT (IU/L)	0.034	0.722	-	-	0.273	0.094	-	-
ALP (IU/L)	0.061	0.742	-	-	0.282	0.227	-	-
Total bilirubin (mg/dL)	0.113	0.241	-	-	0.073	0.329	-	-
Total cholesterol (mg/dL)	0.244	0.081	-	-	0.169	0.081	-	-
Triglycerides (mg/dL)	0.282	0.012	0.273	0.023	0.286	0.047	0.164	0.349
Albumin (g/dL)	−0.261	0.099	-	-	−0.081	0.273	-	-
LDL-c (mg/dL)	0.073	0.541	-	-	0.172	0.075	-	-
HDL-c (mg/dL)	0.294	0.039	0.027	0.411	−0.148	0.021	0.027	0.651
Hypertension	0.193	0.136	-	-	0.193	0.091	-	-
T2DM	0.263	0.029	0.215	0.033	0.075	0.391	-	-

BMI: body mass index, CRP: C-reactive protein, ALT: alanine aminotransferase, AST: aspartate aminotransferase, GGT: gamma-glutamyl transferase, ALP: alkaline phosphatase, LDL-c: low-density lipoprotein cholesterol, HDL-c: high-density lipoprotein cholesterol, T2DM: type 2 diabetes.

## Data Availability

The data presented in this study are available on request from the corresponding author. The data are not publicly available because they are the property of the Institute of Gastroenterology and Hepatology, Iasi, Romania.
